# Repertory of the Back

**Published:** 1899-06

**Authors:** E. H. Wilsey

**Affiliations:** Parkersburg, W. Va.


### REPERTORY OF THE BACK.

E. H. Wilsey, M. D., Parkersburg, W. Va.

Continued from May No., p. 244.

### SACRUM.

**Abdomen**, constant, dull, heavy pain in sacrum and abdomen extending to thighs and legs, *Sepia*.
— pain from abdomen down through back to sacrum, *Armor-sat.*
— pain in sacrum during stool, with distension of abdomen extending into chest, *Carbo-an.*
— menses delayed by five days, with pains in abdomen and sacrum, Sul-ac.
— during the menses, pain in abdomen, straining and pressure, like labor pains, anxious pains in the back and sacrum commencing with tickling, Graph.
— pressive pain in sacrum so that he had to remain bent down (with pressive pain in abdomen), Caust.
— sore pain in abdomen, with pressure toward genitals as in menses and pain in sacrum, Kali-c.
— severe sacral pains with labor-like pains in abdomen and discharge from vagina, Kali-c.
— an occasional stitch from the sacrum through left side of abdomen toward chest, Kali-c.
— pain in sacrum so violent that it drew chest together, with pressure on stomach and constriction of abdomen, Lyco.
— sore pain on inner side of sacrum toward the abdomen, equally when at rest and when in motion, Nat-c.
— frequent pinching, daily in the whole epigastrium, in the sides of the abdomen and toward the sacrum, Nat-m.
— burning pain in abdomen when sitting bowed forward, extending into sacrum and ceasing when he straightens himself, Nitrum.
— ordinary stool after previous pinching and pain in the abdomen and sacrum, Nitrum.
— pressure in the abdomen and on the sacrum as from flatus,

SACRUM.

which also passes off sparingly, affording some relief, Phos.

**Abdomen,** sensation of inflation and at times actual inflation of abdomen, at times pressure; better by moving, with impeded deep respiration or with a bruised pain in sacrum and abdomen, Phos.

— pinching then shooting pains in abdomen and sacrum especially in morning and evening, Nitrum.

**Above,** frequent pain just above sacrum when sitting, Kali-c.

— tickling, fatiguing pain above sacrum, Kali-c.

**Aching** in sacrum in evening. Better by pressure, *Sepia.*

— in sacrum, *Sil.*, *Phyto.*

— and soreness of thighs as if beaten, with aching in sacral bones, *Calc-ph.*

— beating throbbing pain in lumbo-sacral region, Sil.

— after confinement aching pain in sacrum, and very suddenly extends down limbs to knees like in the bone, passing down gradually to ankle and returning again to sacrum, with jerking pains in different parts of the body, Phyto.

— pains in sacrum also down into each buttock, *Helon.*

— prolapsus uteri with dragging aching pain in sacrum, *Helon.*

— in sacrum and knees, Jambos.

— violent aching pain in sacrum at night; better daytime if moving about slowly, but unable to lift anything, *Kali-b.*

— across joints of sacrum, *Kalmia.*

— in sacral region extending down thighs, China.

— in region of left sacro-iliac articulation with crawling in left sole as though it were asleep, *Coloc.*

— severe aching in sacral region from walking a short distance, followed by nausea and lassitude, Con.

— constant aching in sacrum, much worse by walking or stooping forward, Æscul.

— pain in lumbar region and sacrum, especially during exertion in daytime and while sitting, pain sore aching, back not sensitive to touch, *Agaricus.*

### SACRUM.

**Across**, cutting pain across top of sacrum and in left hip, also in muscles just above sacro-iliac symphysis of left side, Gambogia.

— dull pains across loins and sacrum, Lil-tig.

— bruised pain across sacrum and both hips with sensitiveness to touch, Mag-mur.

— stitches transversely across sacrum, Sul.

— severe pain across sacrum and back as if it would break in two, Æscul.

— constant dull pain across hips and sacrum, *Æscul.*

— labor-like pains across sacrum, *Kali-c.*

— aching across joints of sacrum, *Kalmia.*

— intense unbearable pain across supra sacral region extending to right nates and down right sciatic nerve, Lac-can.

— tensive pain from sacrum across left hip, on least motion interfering with walking, Sarsa.

— pain across sacrum with urging to stool, Amm-benz.

**Across,** pressing dragging pain over sacrum, and at same time over hips, burning pressure in sacrum extending across dorsal region and under right scapula like that produced by sewing, *Sepia.*

— sharp stitches across sacrum close above the hips, Nat-m.

— shooting in lumbo-sacral region extending across pelvis to left trochanter on stepping, causing limping, Sul.

**Afternoon**, sprained pain in sacrum above hips in the evening in bed and in afternoon, Sepia.

— weakness of sacrum in afternoon when standing at a desk writing, Lith-c.

— bruised pain in sacrum from morning till afternoon, Mag-c.

**Alternating** with headache, a broken sensation in lumbo-sacral region, worse by sitting, better by walking or standing, Melil.

**Ants**, crawling in sacrum as from ants, Sarsa.

**Anus**, jerking pain extending from left sacrum to anus, Calc.

**Apart**, sacro-iliac synchondroses, feel as if falling apart, wants to be bound tightly, Trill.

### SACRUM.

**Around,** pain in sacrum extends around hips and down right leg, *Bap.*

**Awaking.** pain in sacrum in morning on awaking, she could not remain in bed, she must get up, Nitrum.

— pain in sacrum in morning on awaking extending into left hypochondrium, for several hours, Nitrum.

**Backache,** involves sacrum and hips, Puls.

**Bearing** down on to sacrum, Bell.

— pain in sacrum and bearing down as if parts would be forced out, worse when moving, Secale.

**Bed,** painful throbbing in sacrum evening after going to bed, Nat-m.

— severe itching on sacrum evening while in bed, Nat-m.

— all joints are stiff in morning on rising from bed, especially the shoulders, sacral and hip joints, Staph.

— painless jerks in evening in bed, Sul.

— sits up in bed, leaning forward to avoid contact of bed-clothes, *Lob-i.*

**Beaten,** feeling as if beaten in sacrum, *Nat-m.*

— pain in os sacrum as if beaten in morning, worse stooping, not better by rising again, extending from time to time to renal region, better by walking in open air, worse on right side, Tell.

— feels as if beaten or dislocated in sacrum, *Agari.*

**Beaten,** pains in the back and sacrum as if beaten, Sul.

— aching and soreness of thighs as if beaten, with aching of sacral bones, *Calc-Phos.*

**Beating,** aching, throbbing pain in lumbo-sacral region, *Sil.*

**Belching,** ameliorates pain in back, *Sepia.*

**Bending,** pains in sacrum when bending backward, Con., Mang.

— pain in sacrum only when bending backward, not while at rest, Kali-c.

**Bent,** back and sacrum stiff, cannot be bent; after some exertion in riding, walking, and stooping he can raise himself only slowly and with much difficulty, Lyco.

SACRUM.

**Biting,** burning and sticking as from a mustard plaster between skin and flesh of sacrum in afternoon, Aloe.

— sudden biting on right side of sacrum, Phelland.

**Blow,** in the left hypochondrium pain as after a violent blow, with pain in sacrum often so intense that she could not lie down, followed by leucorrhœa of eight days' standing ; this and the pains in the sacrum only ceased with the appearance of the menses, Nitrum.

— pain in sacrum as if she had received a blow, mornings, Nitrum.

**Bone,** severe pain in sacrum almost only when moving, so that he could hardly walk ; it seems to be in the bone, Nit-ac.

— shooting and tearing in sacral bones, Zinc.

— needle-like pains in the sacrum and like pains in the hip bones, Chimaphila.

**Board,** feels as if lying on a board ; has to change position often, bed feels so hard ; worse in sacrum, *Bapt.*

**Boring,** and burrowing in sacrum and anus, Calad.

— in sacrum, Ledum.

**Break,** severe pain in back and in sacrum as if it would break in two, *Æscul.*

**Breath,** stitching, jerking, piercing in back and sacrum arresting breath, Caustic.

— attacks of stitches in the sacrum that take away the breath, with pain in the head and nape, followed by frequently alternating chill and heat, with anxiety about the scrobiculus cordis until evening, Sulph.

— stitches close above the sacrum when taking a deep breath, Carbo-an.

**Breathing,** sticking in sacrum on breathing, *Merc.*

**Breathe,** painful tension in sacrum so that he cannot breathe deeply, Nit-acid.

**Broken,** drawing pain in sacrum and a sensation as if broken when walking, standing and lying, Carbo-an.

### SACRUM.

**Broken** pain in sacrum as if it were broken, especially sensible to bending backward, Plat.

— pain in sacrum with hard stool, as if it would be broken; with colic as if the bowels would burst, Lyco.

— violent pain in sacrum as if it was broken on moving, Kali-c.

— pain in sacrum as if broken in two when bending and stretching, Mag-m.

— sensation in sacrum in morning as if broken while in bed and on rising she could raise nothing from the ground till 8 or 9 o'clock, then hunger, then cutting in abdomen and diarrhœa which was slimy, Staph.

— pain in sacrum as if broken, anxiety and restlessness and rush of blood to head, *Ars.*

**Bruised** pain in sacrum, Arn. Merc., *Fluor-ac.*, Ginseng.

— severe bruised pain in sacrum chiefly in morning when rising, Kali-c.

— pain in sacrum and small of back, *Nat-s.*

— pain in sacrum when lying on side, Actea-spicata.

— pain in sacrum when walking as if bruised, Hepar.

— pain in sacrum from morning till afternoon, Mag-c.

— violent bruised pain in sacrum from afternoon till evening, Mag-c.

— sensation in sacrum (left) on stooping and on rising, Ver-alb.

— pain in sacral region in evening, Sarsa.

— sensation in hip bones, Ruta.

— digging as if bruised just above sacrum when sitting after a long walk, better by continued walking, returning when standing still or sitting, *Ruta.*

— cramp-like pain in sacrum on pressure, Plat.

— pain in sacrum as if bruised when starting to move after lying down, Dig.

— pain in sacrum equally violent when in rest or in motion, Nat-c.

— pain in sacrum when sitting and when rising up, Nat-m.

— feeling in sacral region, Verat-a.

### SACRUM.

**Bruised** pain in sacrum at night, at 3 A. M., she could not turn over for pain, Nitrum.

— pain in sacrum in every position in evening during menses, Nitrum.

— severe bruised pain in sacrum and coccyx, Sul.

— sacral region and back feel bruised in morning on arising, Callad.

— impeded deep respiration or with a bruised pain in sacrum and abdomen, Phos.

— sensation as from a bruise in sacrum toward evening for several hours, with discharge of leucorrhœa, Caust.

— pain in sacrum as if contracted and bruised, particularly while sitting, less when walking, Mag-c.

— after menses violent pain in sacrum as if bruised during stooping and at other times in the afternoon and evening, Mag-c.

— severe pain and bruised sensation in sacrum and extremities, copious sweat without relief, spasms, Eupat-perf.

**Bruise,** pain as from a bruise in sacrum, Graph, Mag-m.

— pain in sacrum and in back as from a bruise, *Alum.*

**Bubbles**, pain as from inflation in sacrum in A. M. in bed, with a feeling as if great bubbles were accumulating in sacratory, Kali-c.

**Burning** and itching in sacrum above nates, Mag-c.

— in sacrum somewhat to right, a pressive burning, Stann.

— pain in sacrum near the anus, Sul.

— sore or burning pain near sacro-iliac symphysis, Rumex.

— pain in spot above left sacral region extending to first lumbar vertebræ, Mang.

— stitch in sacrum causing one to start, *Mur-acid.*

— itching in sacrum and below right patella, Kali-c.

— itching, worse by scratching on outer part of thigh, on sacrum and hips, succeeded by burning, Mag-m.

— tearing and burning in sacrum and hips afternoon and night, Mag-m.

— pain in spot above sacrum, Phos-ac.

— in os sacrum at 4 P. M., Lachn.

SACRUM.

**Burning** in sacrum, Colch.
— severe burning along sacrum, Coloc.
— pain in sacrum, Sul-ac.
— biting and sticking as from a mustard plaster between skin and flesh of sacrum in P. M., Aloe.
— pressing, dragging pain over sacrum and at same time over hips, a burning pressure in sacrum extending across dorsal region under scapulæ, like that produced by sewing, *Sepia.*

